# Intranasal delivery of BDNF rescues memory deficits in AD11 mice and reduces brain microgliosis

**DOI:** 10.1007/s40520-020-01646-5

**Published:** 2020-07-16

**Authors:** Chiara Braschi, Simona Capsoni, Roberta Narducci, Andrea Poli, Gabriele Sansevero, Rossella Brandi, Lamberto Maffei, Antonino Cattaneo, Nicoletta Berardi

**Affiliations:** 1 Institute of Neuroscience of the CNR, Via G. Moruzzi 1, 56124 Pisa, Italy; 2 Department of Neuroscience, Psychology, Drug Research, Child Health (NEUROFARBA), Florence University, Florence, Italy; 3 Scuola Normale Superiore, Pisa, Italy; 4 Human Physiology Section, Department of Biomedical and Specialty Surgical Sciences, University of Ferrara, Ferrara, Italy; 5 European Brain Research Institute, Rome, Italy; 6 IRCCS Stella Maris, Calambrone, Pisa, Italy

**Keywords:** BDNF, Neurodegeneration, AD11, Alzheimer's disease

## Abstract

A decrease in brain-derived neurotrophic factor (BDNF), a neurotrophin essential for synaptic function, plasticity and neuronal survival, is evident early in the progression of Alzheimer’s disease (AD), being apparent in subjects with mild cognitive impairment or mild AD, and both proBDNF and mature BDNF levels are positively correlated with cognitive measures. BDNF delivery is, therefore, considered of great interest as a potentially useful therapeutic strategy to contrast AD. Invasive BDNF administration has indeed been recently used in animal models of AD with promising results in rescuing memory deficits, synaptic density and cell loss. Here, we tested whether non-invasive intranasal administration of different BDNF concentrations after the onset of cognitive and anatomical deficits (6 months of age) could rescue neuropathological and memory deficits in AD11 mice, a model of NGF deprivation-induced neurodegeneration. In addition to AD hallmarks, we investigated BDNF effects on microglia presence in the brain of AD11 mice, since alterations in microglia activation have been associated with ageing-related cognitive decline and with the progression of neurodegenerative diseases, including AD. We found that intranasal delivery of 42 pmol BDNF (1 μM), but not PBS, was sufficient to completely rescue performance of AD11 mice both in the object recognition test and in the object context test. No further improvement was obtained with 420 pmol (10 μM) BDNF dose. The strong improvement in memory performance in BDNF-treated mice was not accompanied by an amelioration of AD-like pathology, Aβ burden, tau hyperphosphorylation and cholinergic deficit, but there was a dramatic decrease of CD11b immunoreactive brain microglia. These results reinforce the potential therapeutic uses of BDNF in AD and the non-invasive intranasal route as an effective delivery strategy of BDNF to the brain. They also strengthen the connection between neuroinflammation and neurodegenerative dementia and suggest microglia as a possible mediator of BDNF therapeutic actions in the brain.

## Introduction

Alzheimer’s disease (AD) is a progressive neurodegenerative disorder of the central nervous system which is by far the most common cause of dementia in the world and still lacks effective therapeutic strategies [1]. Its neuropathological hallmarks are the accumulation of β amyloid protein (Aβ) and hyperphosphorylated tau, followed by formation of extracellular amyloid plaques and intracellular neurofibrillary tangles [2, 3], together with a deficit of the cholinergic system in the basal forebrain [4].

A decreased expression of the neurotrophin brain-derived neurotrophic factor (BDNF) has been linked to AD. BDNF is a neurotrophin essential for synaptic function, neural plasticity and survival [5]. A decrease in BDNF expression is evident early in the progression of AD, being already apparent in subjects with Mild Cognitive Impairment or mild AD [6–10]. In particular, the decrease in mature BDNF and proBDNF precedes the decline in the activity of acetylcholine biosynthetic enzyme, choline acetyltransferase and both proBDNF and mature BDNF levels are positively correlated with cognitive measures [11].

The involvement of BDNF dysfunction in AD is further suggested by growing evidence showing that ß-amyloid can cause synaptic alterations by disrupting BDNF trafficking and signalling [12], particularly its oligomeric forms may contribute to a reduction of BDNF in animal models of familiar AD [8] and in human in vitro neurons [13]. BDNF content in AD brains seems regulated by specific miRNA, such as miRNA 206 [14] and by peptides such as PACAP [15].

Based on these observations and given the control exerted by BDNF on cortical plasticity and its neuroprotective action, BDNF delivery is considered of great interest as a potentially useful therapeutic strategy for AD. Like other growth factors, BDNF is a polar protein that does not readily cross the blood–brain barrier. To overcome this limit, preclinical studies with BDNF in aged rodents and mouse models over-expressing APP have been performed using invasive gene therapy approaches. These studies showed that BDNF delivered using lentiviral vectors to the entorhinal cortex at early ages delays the onset of learning and memory deficits, increases the expression of presynaptic proteins such as synaptophysin and prevents neuronal loss [16–18]. No effects were evident on Aβ plaque load [16, 18]. However, although promising, the gene therapy approach has two main limits: (1) the invasiveness of the method to deliver the vector into the brain (2) the limited brain area reachable with injections, while more than one region could be positively affected by an increase in BDNF expression. In addition, it remains unclear whether administering BDNF after the onset of cognitive decline would be an efficacious treatment.

In the present study we applied a non-invasive, intranasal method developed by Frey and colleagues [19] to deliver recombinant human BDNF after the onset of memory deficits to AD11 mice, a model of neurodegeneration induced by NGF deprivation. The non-invasive method of intranasal administration has been already used to supply another neurotrophin, NGF and its mutant form “painless“-NGF [19–22], in the brain, rescuing neuropathological markers [21–23] and memory deficits in AD11 mice [20, 21], and in mice over-expressing mutant forms of human APP [23]. AD11 mice express a recombinant version of the anti-NGF antibody αD11; they develop an age-dependent cognitive decline, with the first memory deficits appearing at 4 months of age, and a neurodegeneration which encompasses a comprehensive set of hallmarks of human AD, including the cholinergic deficit and neuroinflammation [24]. Alterations in neuroinflammatory processes and in microglia activation have recently been associated with ageing-related cognitive decline and with the progression in neurodegenerative diseases, including AD [25, 26]. Interestingly, at 1 month of age, AD11 mice show a decreased expression of BDNF mRNA [27].

BDNF intranasal treatment was performed in AD11 mice from 6 to 6.5 months of age, when memory and anatomical deficits are already present. We found that, at this age, BDNF mRNA levels are decreased and that intranasal administration of BDNF could rescue memory deficits and decrease microglial activation.

## Methods

### Animal experimentation

AD11 anti-NGF mice were produced as described [24]. Wild-type (WT) mice were obtained by crossing non transgenic littermates of AD11 mice. Mice were kept under a 12 h dark to light cycle, with food and water ad libitum. BDNF treatment was performed from 6 months of age and experimental analysis was performed on 6.5-month-old mice.

### Ethical approval and compliance with guidelines for experimentation in animal subjects

All experiments have been performed on animals housed at the Institute of Neuroscience of the CNR, Pisa. The Institute has been authorized by the Italian Ministry of Public Health to the use of animals for scientific purposes (Authorization # 129/2000-A, released on December 13, 2000). The Animal Facility currently hosts 450 mice and 420 rats and has a Veterinarian Medical Doctor who takes care of the animals. The local Sanitary Unit controls that all procedures are performed in strict compliance with protocols approved by Italian Ministry of Public Health, and in conformity with the European Communities Council Directive (86/609/EEC, 24 November 1986, OJ L 35812 December 1987), which regulates the use of vertebrate animals in research laboratories.

### BDNF intranasal delivery

BDNF (Harlan) administration was performed on anaesthetized mice as described in De Rosa et al. [20]. Briefly, 2,2,2-tribromethanol (Sigma–Aldrich, St. Louis, Mo) was dissolved in absolute ethanol at the concentration of 1 g/ml and stored at 4 °C in the dark. After dilution in 0.9% NaCl at the final concentration of 1.5%, it was injected i.p. at the dosage of 250 mg/kg of body weight to induce anaesthesia, which followed within 5–10 min after injection. After anaesthesia, mice were laid on their back, with the head in upright position, as described before [19, 20, 22, 28]. A solution of BDNF in 0.1 M phosphate-buffered saline (PBS, pH 7.4) was administered intranasally to AD11 mice at the dose of 12.6, 42 and 420 pmol (0.3, 1 and 10 µM), The solution was delivered 3 μl at a time, alternating the nostrils, with a lapse of 2 min between each administration, for a total of 14 times. During these procedures, mice were kept on their back and the nostrils were always kept open. As control, AD11 mice were treated with PBS. Administrations were repeated for 7 times, at alternate days, for a total of 15 days.

### Behavioural analysis

#### Visual object recognition test (vORT)

The apparatus consisted of a square arena (60 × 60 × 30 cm) constructed in PVC with black walls and white floor. The objects were cubes (12 cm wide) made of transparent Plexiglas that contained the visual patterns to be discriminated. Box and objects were cleaned up between trials to stop the build-up of olfactory cues. The experimental protocol was the same as in De Rosa et al. [20]. Briefly, mice received 3 sessions of 10-min duration in the empty box to help them habituate to the apparatus and test room. Each mouse was then placed in the box and exposed to two cubes with identical visual patterns (sample phase) for 5 min and returned to its cage. After a delay of 1 h and 24 h, respectively, mice were placed back in the box and exposed to a familiar object (visual pattern identical to those in sample phase) and to a novel object (different visual pattern) for 5 min (test phase). Objects were placed in the same locations as in sample phase. Time spent exploring each object was recorded for each animal and for each condition and a discrimination index was calculated:

$${\text{Discrimination}}\;{\text{Index}}\; = \;{{\left[ {\left( {{\text{Exploration}}\;{\text{time}}\;{\text{of}}\;{\text{New}}\;{\text{object}}} \right){-}\left( {{\text{Exploration}}\;{\text{time}}\;{\text{of}}\;{\text{Old}}\;{\text{object}}} \right)} \right]} \mathord{\left/ {\vphantom {{\left[ {\left( {{\text{Exploration}}\;{\text{time}}\;{\text{of}}\;{\text{New}}\;{\text{object}}} \right){-}\left( {{\text{Exploration}}\;{\text{time}}\;{\text{of}}\;{\text{Old}}\;{\text{object}}} \right)} \right]} {\left[ {\left( {{\text{Exploration}}\;{\text{time}}\;{\text{of}}\;{\text{New}}\;{\text{object}}} \right)\; + \;\left( {{\text{Exploration}}\;{\text{time}}\;{\text{of}}\;{\text{Old}}\;{\text{object}}} \right)} \right]}}} \right. \kern-0pt} {\left[ {\left( {{\text{Exploration}}\;{\text{time}}\;{\text{of}}\;{\text{New}}\;{\text{object}}} \right)\; + \;\left( {{\text{Exploration}}\;{\text{time}}\;{\text{of}}\;{\text{Old}}\;{\text{object}}} \right)} \right]}}.$$

#### Object in context test

For the object context test (OCT), two open field arenas (60 × 60 × 30 cm) made of poly vinyl chloride were used. Each arena constituted a different experimental condition (A and B). In condition A, horizontal white stripes were applied on the black walls of the arena. The floor was covered with rough Plexiglas. The experimental protocol was as in De Rosa et al. [20]. In condition B, the arena had grey walls, and the floor was made of Plexiglas. The particular object for a given test was randomly determined, but each object was used for only one experimental condition. The OCT was used to determine whether mice were sensitive to a change in context for a given object. The habituation phase started 2 days before the block of tests and consisted of four sessions. In each session, mice were exposed to both conditions (A and B). In the first and second sessions, mice were placed into the empty arena for 10 min. In the last two sessions, they were allowed to explore the arena for 3 min individually. The OCT was divided into four sample phases and a test phase, each lasting 3 min. The retention interval within the sample phases was 2 min. There was a 5-min interval between the last sample phase and the test phase. In the sample phase, two objects were placed in adjacent corners of the arena; phases 1 and 4 comprised objects A_1_ and A_2_ in environment A, and phases 2 and 3 comprised objects B_1_ and B_2_ in environment B. The test phase was in the same environment as sample phase 4, but one of the objects (A_2_) was replaced by B_2_. In this way, one object was in the same environment as in the sample phase, and the other object was in a different environment from the sample phase. To avoid the eventual preference for one of two environments, half of the mice began the sample phase in environment A with object A_1_ and A_2_ and finished with the same environment with object A_1_ and B_2_ and vice versa.

### Histological analysis

After behavioral analysis, AD11 and WT mice were deeply anaesthetized with chloral hydrate and intracardially perfused with a 4% solution of paraformaldehyde in PBS. Brains were collected and post-fixed in the same solution for 4 h, transferred in 30% sucrose/PBS solution and then sectioned at a sliding freezing microtome (Leica, Wetzlar, Germany). Forty-micrometer sections were collected in 0.05% sodium azide/PBS in 1.5 ml tubes and stored at 4 °C until usage. To detect choline acetyltransferase (ChAT) in basal forebrain neurons, β-amyloid, CD11b in the hippocampus and phosphorylated tau (pTau) in the entorhinal cortex, the following primary antibodies were used: goat anti-ChAT (1:500, Millipore, Billerica, MA); goat anti –NH2 terminus of β-amyloid (1:100; Santa Cruz Biotechnologies, Santa, CA), goat anti-CD11b (1:100, Santa Cruz) and mouse anti-human phosphotau recognizing Ser199 (1:10 clone AT8; Pierce Endogen, Rockford, IL), anti-synaptophysin (1:10, clone SY38, Abcam, Cambridge, UK). The primary antibody signal was detected using the appropriate biotinylated secondary antibody and the avidin horse radish peroxidase system by Vector Laboratories Inc. (Burlingame, CA), with the exception of anti-β-amyloid antibodies that were detected using the avidin-alkaline phosphatase system (Vector Labs).

### Stereology of basal forebrain, hippocampus and cortex

Morphometric analysis was performed using a Nikon microscope (Eclipse 1000, and the morphometry LUCIA program (Laboratory Imaging Ltd., Prague, Czechoslovakia).

The total number of ChAT-positive neurons in the basal forebrain (medial septum plus diagonal band) and nucleus basalis of Meynert (NBM) were calculated according to previous protocol [27], according to the optical fraction method. A similar approach was used to calculate the number of p-tau-positive neurons in the lateral entorhinal cortex and Aβ immunoreactive clusters of dystrophic neurites in the hippocampus, as described [20].

#### RNA extraction

Hippocampus was dissected from the brains of freshly killed AD11 or control mice at 6 months of age. Total RNA was isolated from these brain areas using Trizol (Invitrogen) and DNAse treated by Qiagen columns. Quality and integrity of each sample were checked using the Agilent BioAnalyzer 2100 (Agilent RNA 6000 nano kit): samples with a RNA Integrity Number (RIN) index lower than 8.0 were discarded.

#### Microarray analysis

All the experimental steps involving the labelling, hybridization and washings of the samples were done following the Agilent protocol (http://www.genomics.agilent.com) using an Agilent technologies platform. Aliquots from the same RNA sample, prepared (and pooled) from whole brains of wild-type mice of the same strain (C57BL × SJLF2), were used in all hybridizations as a reference sample, to reduce the experimental variability. The gene expression profiling was performed using a two-colour protocol by Agilent), with a reference experimental design. Control samples and reference sample were always labelled with Cy5 and Cy3, respectively. Cy3 and 5-labelled cRNA were hybridized to Agilent 4 × 44 k whole mouse genome oligonucleotide microarrays (G4122F).

#### Scanning, feature extraction and analysis

Post-hybridization image acquisition was accomplished using the Agilent scanner G2564B, equipped with two lasers (532 nm and 635 nm). Images were analysed by Agilent Feature Extraction. Data filtering was performed in Microsoft Excel by discarding spots close to the background level. Data analysis was performed with Agilent GeneSpring GX, MeV (TIGR) and Microsoft Excel. Every array was normalized by the Lowess algorithm.

#### Western blot analysis

For Western analysis, brains were sonicated (5 times for 30 s on ice) in 5 ml/g (wet weight) of ice-cold extraction buffer (Tris–HCl 50 mM pH 7.5, NACl 150 mM, 1% Igepal, 0.5% Sodium deoxycholate, EDTA 1 mM, 0.1% Sodium Dodecyl Sulfate, protease inhibitor cocktail (Roche)). Homogenates were centrifuged at 16,000×*g* for 45 min at 4 °C. Supernatants were collected and centrifuged again. Samples were analysed by SDS-PAGE (NuPage gels 4–12% Bis–Tris pre cast gels, Thermo Fisher Scientific, Waltham, MA) and Western blot using a mouse monoclonal antibody (clone M2F6, Enzo Life Sciences, Rome, Italy). The intensities of the immunoreactive bands were quantified and analysed using the National Institutes of Health (NIH) image analysis program (NIH IMAGEJ v. 4.2) after normalizing for protein content and evaluated by the intensity of the GAPDH band obtained after incubation with mAb 6C5 (Fitzgerald Industries International, Acton, MA).

### Statistical analysis

All data have been analysed with Sigma Stat statistical package. Where post hoc comparisons were necessary, we have used the suggested post hoc tests.

To compare performance in the vORT and in the OCT, we have used a paired *t* test (or Signed Rank test if the data were not normally distributed) to analyse differences between the exploration time of the new and the familiar object for each group and each retention interval, a two-way repeated measure (RM) ANOVA to analyse the pre-treatment and post treatment performance for each retention interval and a two-way RM ANOVA, group × interval to analyse the performance of WT, AD11 PBS and AD11 BDNF pre- and post-treatment. To analyse differences in immunohistochemistry data between WT, AD11 treated with PBS and AD11 treated with BDNF, one-way ANOVA was performed. Differentially expressed mRNAs were identified by ANOVA and by Significance analysis of microarrays (SAM). Significance levels for post hoc analysis, *p* < 0.05.

## Results

### BDNF expression in 6-month-old AD11 mice

Since an early decrease in BDNF expression was previously found, by microarray analysis, in 1-month-old AD11 mice [21] we tested whether BDNF mRNA was also decreased at 6 months, the age chosen for the BDNF treatment. At 6 months of age, AD11 mice already show a strong deficit in visual recognition memory and cholinergic deficits [22], amyloid-β (Aβ) clusters appear in proximity of dystrophic neurites in the hippocampus and hyperphosphorylated tau are already detectable [23]. We found that BDNF mRNA is still down-regulated in AD11 hippocampus (0.62 linear scale fold change), further reinforcing the rationale for a BDNF treatment.

### Intranasal treatment with a low dose of BDNF (42 pmol)

We first explored the feasibility of a non-invasive intranasal delivery of BDNF by nasal administration of a low dose of BDNF (42 pmol/administration) to AD11 mice, from 6 to 6.5 months of age. PBS-treated AD11 mice were used as controls. AD11 mice were randomly assigned to PBS (AD11-PBS) or BDNF (AD11-BDNF) treatment groups. No difference in body weight was found at the end of the treatment between AD11-BDNF and PBS mice (Wilcoxon Signed Rank Test, *p* = 0.50 for BDNF-treated mice, pre-treatment and post-treatment weight 26 and 25.5 g, respectively, interquartile range [22–30 g] in both cases; Paired *t* test, *p* = 0.48 for PBS-treated mice, pre-treatment and post-treatment weight 27.4 ± 2.7 and 27 ± 2.4 g, respectively).

#### vORT

The tests started at 6.5 months of age, after the end of the intranasal treatment. Performance in the ORT was assessed before and after treatment at two retention intervals, 1 h and 24 h, using the protocol employed in Berardi et al. [29]. No difference was observed between the performance of the two groups before the treatment, and both resulted impaired with respect to WT mice. Treatment with 42 pmol (1 µM) BDNF was effective in improving performance of AD11 mice and the improvement was detectable in both the exploration time and the discrimination index.

Longer exploration of the new object during the test phase indicates that mice have learned about the object during the sample phase and express the normal consequence of visual recognition memory, namely preference for the new object. As expected, there was no differential exploration of the new with respect to the familiar object for AD11 mice in the test performed before the treatment: neither for the animals to be treated with BDNF (*N* = 8, mean exploration time 12 ± 3 s for the new and 15 ± 3 for the familiar object at 1 h interval; 13.5 ± 4 s for the new and 12 ± 3 s for the familiar object at 24 h) nor for those to be treated with PBS (*N* = 8, mean exploration time 13 ± 2 s for the new and 14 ± 2 s for the familiar object at 1 h interval; 12 ± 2 s for the new and 10 ± 2 s for the familiar at 24 h), (paired *t* test, *p* > 0.05). At the end of the treatment, AD11-BDNF mice explored the new object for a significantly longer time than the familiar one both at 1-h and 24-h intervals (5 ± 1.5 s for the new and 2 ± 0.9 s for the familiar object at 1 h and 4 ± 1 s for the new and 2 ± 0.5 s for the familiar object at 24 h, paired *t* test, *p* = 0.04 at 1 h and 0.016 at 24 h). On the contrary, AD11-PBS mice did not show a differential exploration of the new object (7 ± 1 s for the new and 6 ± 2 s for the familiar object at 1 h and 9 ± 1 s for the new and 8.5 ± 2 s for the familiar object at 24 h, paired *t* test, *p* = 0.33 at 1 h and 0.5 at 24 h).

**Fig. 1:**
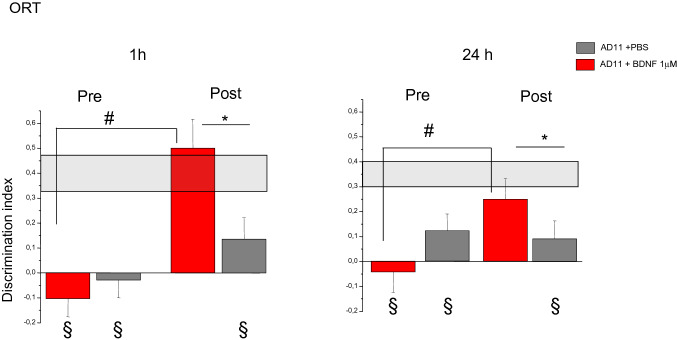
1 μM BDNF Intranasal treatment is sufficient to rescue AD11 mice performance in the object recognition test (ORT). At both 1-h and 2–4 h retention intervals, there is no significant difference in discrimination index between AD11-PBS and Ad11-BDNF mice before treatment, while, after treatment, BDNF treated mice perform significantly better than PBS treated mice (Two way RM ANOVA, pre-post × treatment type; at 1-h interval, factor treatment *p* < 0.001, interaction *p* = 0.02, mean discrimination index of AD11 BDNF vs PBS groups pre-treatment: *p* > 0.05; post-treatment: *p* < 0.05 (asterisk symbol); factor pre-post significant for BDNF, (*p* < 0.05, gate symbol), but not for PBS treated AD11 mice, *p* > 0.05, post hoc Holm–Sidak method; at 24-h interval, factor pre-post *p* = 0.268, interaction *p* = 0.005, AD11 BDNF vs PBS groups pre-treatment: *p* > 0.05; post-treatment: *p* < 0.05 (asterisk symbol), factor pre-post significant for BDNF, (*p* < 0.05, gate symbol), but not for PBS treated mice, *p* > 0.05, post hoc Holm–Sidak method). At both retention intervals, before treatment AD11 BDNF and PBS mice perform significantly worse than WT mice (shaded rectangle, range of performance of WT mice (mean ± sem); Two way RM ANOVA, group × retention interval, factor group *p* < 0.001, WT mice differ both from AD11-BDNF and AD11-PBS, Holm–Sidak post hoc test, *p* < 0.05, § symbol denotes significant difference). After treatment AD11-PBS, but not AD11-BDNF, differs from WT mice (Two way RM ANOVA, group × retention interval, factor group *p* = 0.005, WT vs Ad11-PBS and WT vs Ad11-BDNF, Holm–Sidak post hoc test, *p* < 0.05 and *p* > 0.005, respectively)

Higher discrimination indexes reflect a longer exploration time of the familiar object in the test phase. Discrimination index of AD11-BDNF (*N* = 8) and AD11-PBS mice (*N* = 8) did not differ before treatment, while it differed after the end of the treatment, with BDNF-treated animals performing significantly better than those treated with PBS; moreover, AD11-BDNF mice showed improved performances at the end of the treatment in comparison with the pre-treatment tests(Fig. 1) (Two way RM ANOVA, pre-post-treatment × treatment type at 1-h interval, factor pre-post *p* = 0.137, factor treatment *p* < 0.001, interaction *p* = 0.02, mean discrimination index of AD11 BDNF vs PBS groups *p* > 0.05 pre-treatment and *p* < 0.05 post-treatment; mean discrimination index of AD11 BDNF mice post-treatment significantly higher than pre-treatment, *p* < 0.05; performance of AD11 PBS animals pre- and post- treatment not significantly different, *p* > 0.05, post hoc Holm–Sidak method; at 24-h interval, factor pre-post *p* = 0.268, interaction *p* = 0.005, mean discrimination index of AD11 BDNF vs PBS groups *p* > 0.05 pre-treatment and *p* < 0.05 post-treatment; mean discrimination index of AD11 BDNF mice post-treatment significantly higher than pre-treatment, *p* < 0.05; performance of AD11 PBS animals pre- and post-treatment not significantly different, *p* > 0.05, post hoc Holm–Sidak method).

We also performed a comparison of the performance of AD11 mice and WT mice of the same age (WT, *N* = 14, mean discrimination index and standard error at 1 h 0.4 ± 0.07, at 24 h 0.35 ± 0.05).

As expected [20, 29], before the treatment, the performance of AD11-BDNF and AD11-PBS mice differed from that of WT mice at both 1- and 24-h interval, with a higher discrimination index in WT mice (Two-way RM ANOVA, group × retention interval, factor group *p* < 0.001, WT differ both from AD11 to be treated with BDNF and PBS, while the latter do not, Holm–Sidak post hoc test, *p* < 0.05). Past the end of the treatment, performance of AD11-PBS animals differed from that of both WT and AD11-BDNF mice at both 1- and 24-h interval; performance of AD11-BDNF animals did not differ from that of WT mice (Two way RM ANOVA, group × retention interval, factor group *p* = 0.005, WT and AD11 mice treated with BDNF differ from AD11 mice treated with PBS, while WT and AD11 mice treated with BDNF do not, Holm–Sidak post hoc test, *p* < 0.05).

Thus, treatment with 42 pmol BDNF was sufficient to fully rescue the performance of 6.5-month-old AD11 mice that are already impaired in object recognition memory, to the point that BDNF treated mice perform as well as WT mice.

#### OCT

In humans performance in recognition memory tests is often better when learning and test occur in the same context, than if learning and test contexts are different [30, 31]. Context-dependency effects have been reported on tests of object recognition memory in rats [32], which suggests that, as in humans, recognition processes in nonhuman animals are modulated by memory of contextual information. Performance in this test is strongly dependent on hippocampus and hippocampal plasticity [33, 34].

To assess whether the 42 pmol dose of BDNF was effective in improving performance in recognition of objects in a context-dependent manner we performed the object in context test (OCT) [32–34] using the protocol described in De Rosa et al. [20] but employing a retention interval of 24 h. To do so, AD11 mice (*N* = 6) were tested before and after treatment.

**Fig. 2:**
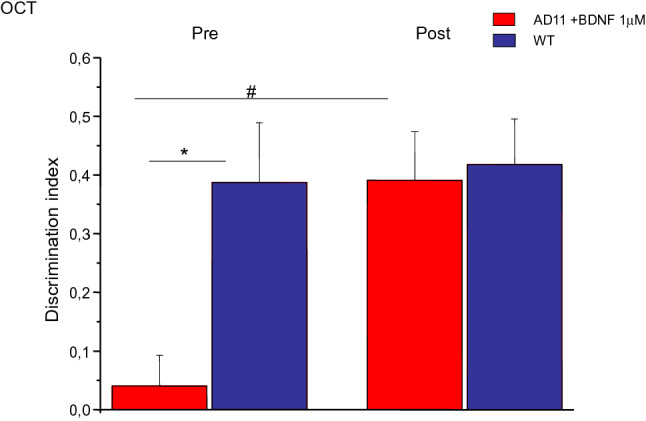
Intranasal treatment with 1 μM BDNF rescues performance of AD11 mice in a memory test involving recognition of an object in a given context. Performance of AD11 mice significantly differed form WT performance before being treated with BDNF while it did not after treatment; performance of WT mice did not significantly change between the first and the second assessment (Two way RM ANOVA, factor pre-post *p* < 0.025, WT vs BDNF AD11 mice differ in the assessment pre treatment (*p* < 0.05, asterisk symbol) but do not differ after treatment; pre-treatment performance of AD11 mice differ from that post BDNF treatment (*p* < 0.05, gate symbol); WT pre-treatment performance does not differ from that post-treatment, Holm–Sidak post hoc test)

We found that there was a significant improvement of performance after treatment with respect to before (Fig. 2) (paired *t* test, *p* = 0.007). Differential exploration of the object novel for the context with respect to the one familiar for the context was not present before treatment (mean exploration of novel object 12 ± 3 s, mean exploration of familiar object 11 ± 3 s, paired *t* test *p* = 0.428) while it was present past the end of the treatment (mean exploration of novel object 7 ± 2 s, mean exploration of familiar object 2.6 ± 1 s, paired *t* test *p* = 0.018). Since De Rosa et al. [20] have already shown that intranasal PBS treatment in AD11 animals of the same age as the ones we employed did not improve performance in the OCT at a much shorter retention interval (5 min) we decided not to include a PBS-treated AD11 group.

The comparison with WT (Fig. 2) shows that AD11-BDNF mice significantly differed from WT (*N* = 10) before being treated with BDNF, while they did not after treatment. The performance of WT mice did not significantly change between the first and the second assessments (Two-way RM ANOVA, factor pre-post *p* < 0.025, WT vs BDNF AD11 mice differ in the assessment pre-treatment but do not differ after treatment; performance of BDNF AD11 mice pre-treatment differ from that post-treatment; WT pre-treatment performance does not differ from that post-treatment, Holm–Sidak post hoc test, *p* < 0.05).

Intranasal treatment with 42 pmol BDNF was, therefore, sufficient to rescue performance of AD11 mice also in the OCT test with retention interval of 24 h.

Thus, treatment with 42 pmol BDNF was sufficient to completely rescue performance of AD11 mice both in the object recognition test and in the object context test.

We further tried whether a lower dose of BDNF (12.6 pmol, 0.3 μM) could be effective. We found that, despite a positive trend towards a better performance (pre-treatment vs post-treatment) both in the ORT and in the OCT test, the improvement was not significant (data not shown).

### Immunohistochemistry for AD hallmarks

**Fig. 3:**
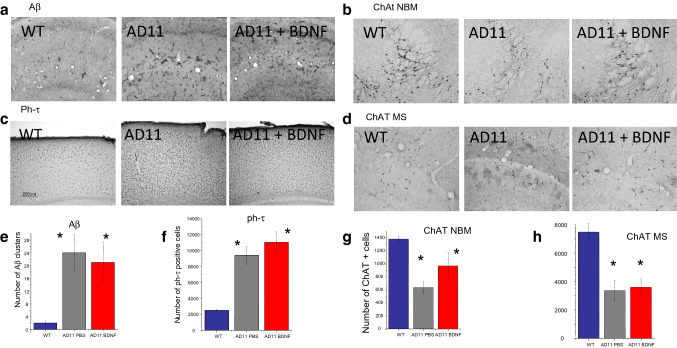
**a**–**d** Examples of staining for Aβ in the hippocampus (**a**), ChAt in the nucleus basalis of Meynert (NBM) (**b**), phospho tau in (ph-τ) the enthorinal cortex (**c**) and ChAt in the medial septum (MS) of a WT, a control AD11 and a BDNF treated AD11 mouse. **e**–**h**, quantification of the number of Aβ clusters (**e**), number of cells positive for phospho-τ (**f**), number of cells positive for ChAt in the NBM (**g**) and MS (**h**) in WT, PBS treated and BDNF treated AD11 mice. For all panels, WT mice differ from AD11 control and BDNF treated mice; the latter two do not differ (One way ANOVA, factor genotype *p* < 0.05, post hoc Tukey’s test). Asterisks denote significant difference with respect to WT mice

We analysed the presence of AD-like neurodegeneration in the hippocampus and cortex of AD11 mice treated with 42 pmol BDNF (*N* = 3) or PBS (*N* = 3) and of 3 WT mice. We found no statistical difference between BDNF-treated AD11 mice and PBS-treated mice, when the number of Aβ clusters was analysed (Fig. 3a and e, mean number and standard error: WT mice, 2 ± 0.7, AD11 mice treated with BDNF, 21 ± 6.5, AD11 mice treated with PBS, 24 ± 5.71; One-way ANOVA, *p* < 0.05, WT vs BDNF treated AD11 mice, *p* < 0.05; WT vs AD11 mice treated with PBS, *p* < 0.05; AD11 mice treated with BDNF vs AD11 mice treated with PBS, *p* = 1.00, post hoc Tukey’s test). Similarly, BDNF treatment was not effective in reducing the cholinergic deficit, neither in the medial septum (MS/DBH) (Fig. 3d and h, ChAT positive cells, mean number and standard error: WT mice, 7479 ± 606, AD11 mice treated with BDNF, 3602 ± 636, AD11 mice treated with PBS, 3363 ± 714; One-way ANOVA, *p* < 0.05, WT vs BDNF-treated AD11 mice, *p* < 0.05; WT vs AD11 mice treated with PBS, *p* < 0.05; AD11 mice treated with BDNF vs AD11 mice treated with PBS, *p* = 1.00, post hoc Tukey’s test) nor in the nucleus basalis of Meynert (NBM) (Fig. 3b and g, ChAT positive cells, mean number and standard error: WT mice, 1382 ± 48, AD11 mice treated with BDNF, 970 ± 202, AD11 mice treated with PBS, 636 ± 94; One-way ANOVA, *p* < 0.05, WT vs BDNF-treated AD11 mice, *p* < 0.05; WT vs AD11 mice treated with PBS, *p* < 0.05; AD11 mice treated with BDNF vs AD11 mice treated with PBS, *p* = 0.248, post hoc Tukey’s test). Also the presence of hyperphosphorylated tau in the cortex of AD11 mice was not affected by BDNF treatment (Fig. 3, c and f, phospho-tau positive cells, mean number and standard error: WT mice, 2498 ± 129, AD11 mice treated with BDNF, 11040 ± 1350, AD11 mice treated with PBS, 9400 ± 1110; One way ANOVA, *p* < 0.05, WT vs BDNF treated AD11 mice, *p* < 0.05; WT vs AD11 mice treated with PBS, *p* < 0.05; AD11 mice treated with BDNF vs AD11 mice treated with PBS, *p* = 0.74, post hoc Tukey’s test).

Thus, the strong improvement in memory performance in BDNF-treated mice is not accompanied by an amelioration of AD-like neuropathology.

### Dendritic and synaptic markers

One of the first deficit present in AD brains is a synaptic failure [35]. Likewise, in AD animal models there is an early loss of synaptic density and spines [36]. Genetic BDNF delivery has been shown to increase synaptophysin expression in AD mouse models [16] and neuronal stem cell transplant seems to achieve the same effect via BDNF [17]. We examined whether intranasal BDNF delivery in AD11 mice could affect synaptophysin expression; in addition we examined expression of drebrin, a dendritic spine protein.

**Fig. 4:**
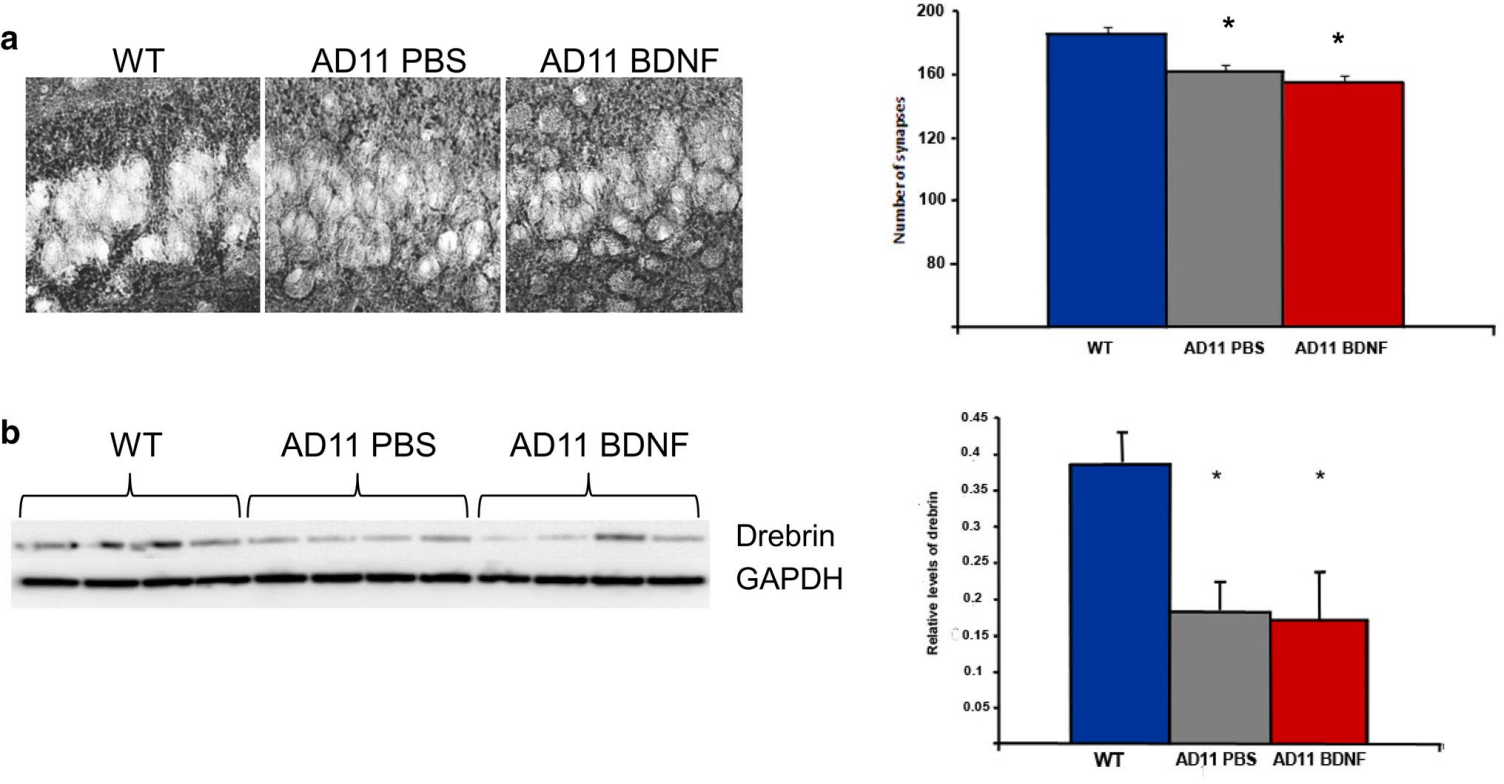
Synaptophysin and Drebrin expression are not rescued after intranasal treatment with BDNF. **a** Immonohistochemistry for synaptophysin and the relative quantification. **b** Western blot and quantification of drebrin expression in brain extracts from WT (lanes 1–4); AD11 mice treated with PBS (lanes 5–8) and AD11 mice treated with 1 µM BDNF (lanes 9–12). Bars are representative of mean ± sem

We found that BDNF delivery did not normalize the reduction in synaptophysin (Fig. 4a) and drebrin expression (Fig. 4b) evident in AD11 mice.

### Immunohistochemistry for activated microglia

**Fig. 5:**
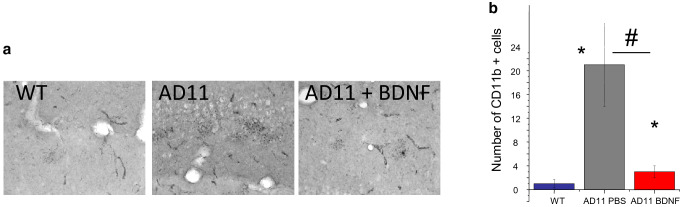
Decreased microgliosis in AD11 mice after treatment with BDNF. **a** Examples of staining for CD11b in the cortex of a WT, a control AD11 and a BDNF treated mouse. **b** Quantification of the number of CD11b positive cells in the hippocampus: WT mice differ from AD11 control and BDNF treated mice (asterisks symbols), BDNF treated AD11 mice significantly differ from control AD11 mice (gate symbol) (One way ANOVA, *p* < 0.05, WT vs BDNF treated AD11 mice, *p* < 0.05; WT vs AD11 mice treated with PBS, *p* < 0.05; AD11 mice treated with BDNF vs AD11 mice treated with PBS, *p* = 0.032, post hoc Tukey’s test)

Microglia was immunostained with and CD11b antibodies [37]. When the number of CD11b-positive cell was analysed, we found that CD11b-positive microglia (Fig. 5a, b) was significantly reduced in AD11 mice treated with BDNF with respect to PBS-treated AD11 mice (mean number and standard error: WT mice, 1 ± 0.7, AD11 mice treated with BDNF, 3 ± 1.08, AD11 mice treated with PBS, 21 ± 7.17. One-way ANOVA, *p* < 0.05, WT vs BDNF-treated AD11 mice, *p* < 0.05; WT vs AD11 mice treated with PBS, *p* < 0.05; AD11 mice treated with BDNF vs AD11 mice treated with PBS, *p* = 0.032, post hoc Tukey’s test).

### Intranasal treatment with a high dose of BDNF (420 pmol)

To assess whether higher doses of BDNF could be beneficial not only in rescuing the memory deficits in AD11 mice but also in ameliorating the AD-like pathology, we treated a group of 6-month-old mice with 420 pmol of BDNF (10 μM concentration). The treatment protocol was identical to that employed for the lower dose and animals were tested in the ORT before and after treatment. As controls, AD11 mice treated with PBS were employed, the same used as controls for the 42 pmol. No difference in body weight was evident after intranasal treatment in AD11 mice, both PBS- and BDNF-treated (Paired *t* test, *p* = 1.00 for BDNF-treated mice, pre-treatment and post-treatment weight 27 ± 3.2 and 27 ± 3 g, respectively; Paired *t* test, *p* = 0.42 for PBS-treated mice pre-treatment and post-treatment weight 26.3 ± 3.2 and 26.7 ± 3.2 g, respectively).

**Fig. 6:**
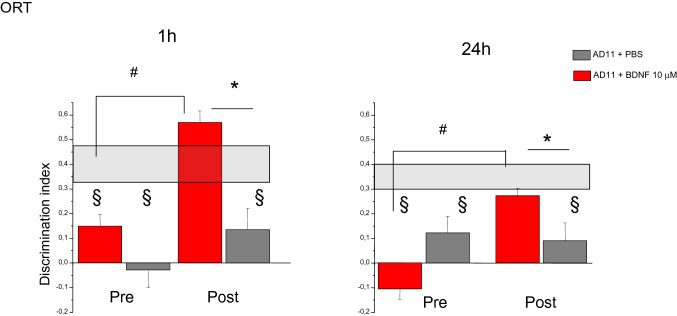
Intranasal treatment with 10 μM BDNF rescues performance of AD11 mice in the ORT with retention intervals of 1 h (1 h) and 24 h (24 h) before (pre) and after (post) BDNF 10 μM or control (PBS) treatment. Shaded rectangle, range of performance of WT mice (mean ± sem). For both retention intervals, there is no significant difference in discrimination index between AD11 mice to be treated with BPS or BDNF before treatment while, after treatment, BDNF treated mice perform significantly better than PBS treated mice (Two way RM ANOVA, pre-post treatment × treatment type; at 1 h interval, factor treatment *p* = 0.044, factor pre-post *p* = 0.007, mean discrimination index of AD11 BDNF vs PBS groups *p* > 0.05 pre-treatment and *p* < 0.05 post-treatment (asterisk symbol); factor pre-post significant for BDNF, (*p* < 0.05, gate symbol), but not for PBS treated AD11 mice, *p* > 0.05, post hoc Holm–Sidak method; at 24 h interval, factor pre-post *p* = 0.015, interaction *p* = 0.007, mean discrimination index of AD11 BDNF vs PBS groups *p* > 0.05 pre treatment and *p* < 0.05 (asterisk symbol) post-treatment, factor pre-post significant for BDNF, (*p* < 0.05, gate symbol), but not for PBS treated AD11 mice, *p* > 0.05, post hoc Holm–Sidak method). Performance of AD11 mice to be treated with BDNF or PBS differs from that of WT mice both at 1-h and 24-h interval, (Two way RM ANOVA, group × retention interval, factor group *p* < 0.001, WT differ both from AD11 to be treated with BDNF and PBS (*p* < 0.05, § symbol), while the latter do not differ between themselves, Holm–Sidak post hoc test). After treatment, performance of AD11 mice treated with PBS still differs from that of WT mice and AD11 mice treated with BDNF both at 1-h and 24-h interval, performance of AD11 mice treated with BDNF does not (Two way RM ANOVA, group × retention interval, factor group *p* = 0.017, WT and AD11 mice treated with BDNF differ from AD11 mice treated with PBS, while WT and AD11 mice treated with BDNF do not differ between themselves, Holm–Sidak post hoc test, the § symbol denotes significant difference with respect to WT mice performance)

As expected, BDNF treatment was effective in improving performance in the vORT at both retention intervals (Fig. 6). There was no differential exploration of the new with respect to the familiar object at the test performed before treatment, neither for the AD11 mice to be treated with BDNF (*N* = 9) (17.3 ± 4 s for the new and 15.1 ± 4 for the familiar object at 1 h interval; 13.1 ± 3 s for the new and 16 ± 4 s for the familiar object at 24 hs) nor for those to be treated with PBS (*N* = 8) (13 ± 2 s for the new and 14 ± 2 s for the familiar object at 1-h interval; 12 ± 2 s for the new and 10 ± 2 s for the familiar at 24h), (paired *t* test, *p* > 0.05). After treatment, BDNF-treated AD11 mice explored the new object for a significantly longer time than the familiar one both at 1- and 24-h intervals (11 ± 2 s for the new and 3 ± 0.5 s for the familiar object at 1 h and 9 ± 1 s for the new and 4 ± 0.6 s for the familiar object at 24 h, paired *t* test, *p* < 0.03 at 1 h and 0.002 at 24 h). On the contrary, PBS-treated AD11 mice did not show a differential exploration of the new object (7 ± 1 s for the new and 6 ± 2 s for the familiar object at 1 h and 9 ± 1 s for the new and 8.5 ± 2 s for the familiar object at 24 h, paired *t* test, *p* = 0.33 at 1 h and 0.5 at 24 h).

Discrimination index of AD11 mice to be treated with BDNF or PBS did not differ before treatment; those of AD11 mice treated with BDNF significantly increased after treatment, while those of PBS-treated animals did not (Two-way RM ANOVA, pre-post-treatment × treatment type at 1-h interval, factor pre-post *p* < 0.007, factor treatment *p* = 0.044, mean discrimination index of BDNF vs PBS groups *p* > 0.05 pre-treatment and *p* < 0.05 post-treatment; mean discrimination index of BDNF animals post-treatment significantly higher than pre-treatment, *p* < 0.05; performance of PBS animals pre- and post-treatment is not significantly different, *p* > 0.05, post hoc Holm–Sidak method; at 24-h interval, factor pre-post 0.015, interaction *p* = 0.007, mean discrimination index of BDNF vs PBS groups *p* > 0.05 pre-treatment and *p* < 0.05 post-treatment; mean discrimination index of BDNF animals post treatment significantly higher than pre-treatment, *p* < 0.05; performance of PBS animals pre- and post- treatment are not significantly different, *p* > 0.05, post hoc Holm–Sidak method).

We also performed a comparison of the performance of AD11 mice and WT mice of the same age (*N* = 14, same animals used for the comparison with the lower BDNF dose, mean discrimination index and standard error at 1 h 0.4 ± 0.07, at 24 h 0.35 ± 0.05). Performance of AD11 mice to be treated with BDNF or PBS differed from that of WT mice both at 1- and 24-h interval, with a higher discrimination index in WT mice (Two-way RM ANOVA, group × retention interval, factor group *p* < 0.001, WT differ both from AD11 to be treated with BDNF and PBS, while the latter do not, Holm–Sidak post hoc test, *p* < 0.05). In the comparison with data obtained after treatment, performance of AD11 mice treated with PBS differed from that of WT mice and AD11 mice treated with BDNF both at 1- and 24-h interval; performance of AD11 mice treated with BDNF did not differ from that of WT mice (Two-way RM ANOVA, group × retention interval, factor group *p* = 0.017, WT and AD11 mice treated with BDNF differ from AD11 mice treated with PBS, while WT and AD11 mice treated with BDNF do not, Holm–Sidak post hoc test, *p* < 0.05).

Thus, BDNF treatment at a higher dose has comparable effects to those at the lower dose on behavioural performance. This was the case also for the anatomical data.

We analysed the presence of Aβ clusters in the hippocampus of AD11 mice treated with BDNF (*N* = 5) or PBS (*N* = 6) and of 6 WT mice. We found that both AD11 mice treated with BDNF and PBS showed a significantly higher percentage of hippocampal area occupied by clusters of Aβ-positive cells than WT mice (mean number and standard error: WT mice, 6.4 ± 2, AD11 mice treated with BDNF, 25 ± 1.5, AD11 mice treated with PBS, 21 ± 1.5. One-way ANOVA, *p* < 0.001, WT vs BDNF treated AD11 mice, *p* < 0.001; WT vs AD11 mice treated with PBS, *p* < 0.001; AD11 mice treated with BDNF vs AD11 mice treated with PBS, *p* = 0.247, post hoc Tukey’s test). Similarly, BDNF treatment was not effective in reducing the cholinergic deficit in the medial septum/diagonal band of Broca (mean number and standard error: WT mice, 6353 ± 562, AD11 mice treated with BDNF, 2899 ± 322, AD11 mice treated with PBS, 3537 ± 152. One-way ANOVA, *p* < 0.001, WT vs BDNF treated AD11 mice, *p* < 0.001; WT vs AD11 mice treated with PBS, *p* < 0.001; AD11 mice treated with BDNF vs AD11 mice treated with PBS, *p* > 0.05, post hoc Tukey’s test) or the presence of hyperphosphorylated tau in the cortex of AD11 mice (mean number and standard error: WT mice, 3031 ± 633, AD11 mice treated with BDNF, 11,007 ± 1886, AD11 mice treated with PBS, 6827 ± 1212. One-way ANOVA, *p* < 0.001, WT vs BDNF treated AD11 mice, *p* < 0.001; WT vs AD11 mice treated with PBS, *p* < 0.001; AD11 mice treated with BDNF vs AD11 mice treated with PBS, *p* > 0.05, post hoc Tukey’s test, although a non significant trend towards an increase in the number of ChAT positive neurons in the Nucleus basalis of Meynert was found (mean number and standard error: WT mice, 1232 ± 141, AD11 mice treated with BDNF, 786 ± 12, AD11 mice treated with PBS, 688 ± 191. One-way ANOVA, *p* < 0.001, WT vs BDNF treated AD11 mice, *p* < 0.001; WT vs AD11 mice treated with PBS, *p* < 0.001; AD11 mice treated with BDNF vs AD11 mice treated with PBS, *p* > 0.05, post hoc Tukey’s test).

It is possible to conclude that, also with this level of neurotrophin, the behavioural improvement was not accompanied by a reduction in the amount and number of Aβ clusters or in the cholinergic deficits and tau pathology.

We do not have information on Cd11b staining for the higher BDNF dose; this is a limit of our paper that does not allow us to assess whether a higher neurotrophin dose led to a lower microglia activation.

## Discussion

The Neurotrophin BDNF has been widely implicated in neurological disorders and in particular in AD [38, 39]. There is, therefore, a significant interest in exploring its therapeutic potential and in finding ways to facilitate its delivery to the brain in a non-invasive way. In AD, the role of BDNF in the pathogenesis of the neurodegeneration and the potential use of BDNF as a therapeutic agent are still under debate. Indeed, while it has been shown that the expression of BDNF mRNA and protein are decreased in the cortex and hippocampus of AD patients [9, 11, 40] as a consequence of Aβ accumulation [41–43], it has been also suggested that BDNF itself might influence AD neuropathology. In particular, the BDNF polymorphisms Val66Met and Cys270Thr have been associated with the risk of developing AD, although in a still unclear manner [44–49]. In addition, BDNF seems to be protective against Aβ neurotoxicity and tau hyperphosphorylation in vitro [50, 51]. However, the rationale for a clinical application of BDNF is mainly based on its activity in synaptic repair, since this neurotrophin is able to regulate synaptic growth and plasticity [5]. Interestingly, recent results show that BDNF deficiency in 3xTg mice does not affect Aβ and tau pathology [52] while early BDNF gene delivery in J20 amyloid over-expressing transgenic mice reversed synapse loss and improved learning and memory [38], but neither affected neuronal number nor amyloid plaque density [18, 38].

Unfortunately, the viral approach to deliver BDNF to the brain of transgenic mice is combined by intrinsic limits linked to the invasiveness of the method and to the fact that the integration of the lentiviral vector is limited to the site of injection. For this reason, several brain areas would not be exposed to the neuroprotective action of BDNF. Thus, the delivery of BDNF to the brain in a non-invasive and widespread manner remains a big challenge. Although its early delivery appears a preventive approach, the BDNF potential in rescuing already existing cognitive deficits still remains an open question.

In this study, we provide a feasibility proof-of-principle of a non-invasive intranasal administration of BDNF and we report the effects of intranasal BDNF on the neurodegeneration observed in 6.5-month old AD11 anti-NGF mice [24], after neurodegeneration has already progressed significantly and cognitive deficits are apparent. This mouse model was chosen on the basis of its progressive neurodegeneration which encompasses a comprehensive set of hallmarks of human AD [53] and for the fact that BDNF mRNA is also precociously decreased in the brain [27].

Given our previous demonstration that NGF reaches the mouse brain in pharmacologically and therapeutically relevant concentrations via the nose-to-brain route [19], we chose to deliver BDNF using the intranasal route. Delivery of peptides to the brain via the olfactory pathway (see [54] and [55] for review) is currently successfully used in pilot studies to deliver candidate drugs for AD clinical trials such as insulin [56, 57]. In rats, intranasally delivered BDNF reaches the brain via the olfactory and the trigeminal nerves and reaches picomolar concentrations in several brain areas, including the hippocampus and, to a lesser extent, the cerebral cortex [58]. BDNF has been successfully delivered to the brain with the intranasal route to improve stress conditions [59] and to protect from ischemic insult [60].

Here we found that two doses of BDNF (42 and 420 pmol, 1 and 10 μM in the delivery solution, respectively) were effective in improving memory performance in tests, ORT and OCT, strongly linked to Medial Temporal Lobe function, fully rescuing performance to the levels measured in non transgenic mice. The discrimination index of PBS-treated AD11 animals, on the contrary, showed no significant variation between the pre- and the post-treatment assessments, indicating that neither the intranasal treatment per se or the repetition of the test could be responsible for the improvement in performance of BDNF treated mice.

BDNF is known to affect energy balance and body weight [61, 62]. The analysis of BDNF-treated mice did not show loss of body weight, suggesting that, at these doses, the intranasal treatment does not induce gross adverse effects. However, due to the large spectrum of activity of BDNF, a more detailed analysis would be required. In this view, the full efficacy already observed with the lower dose allows reducing the risks of side-effects.

The histological analysis of the brain indicates that BDNF administration does not affect Aβ accumulation and tau hyperphosphorylation in AD11 mice. The fact that the intranasal treatment with BDNF improves memory deficits without decreasing Aβ accumulation is not surprising. In other mouse models of AD, BDNF administration does not alter Aβ or tau pathology, but improves cognitive deficit by acting on synaptic plasticity parameters, such as synaptic density [16, 17, 38]. In these first papers amyloid oligomers, known to disrupt synaptic plasticity and learning even in absence of deposits or plaques [63], were not assessed. More recently, a direct assessment of amyloid oligomers following trkB reduction was performed in 5xFAD mice [64] finding that BDNF signalling reduction exacerbates manifestation of hippocampal mnemonic and signalling dysfunctions in early AD without affecting Aβ content and in particular Aβ oligomers. Therefore, a direct action of BDNF on amyloidogenic pathways seems unlikely. This is further confirmed by recent results [65], showing that BDNF intranasal delivery in 5xFAD adult mice does not reduce plaque load.

Several targets can be taken into consideration. In this study we found only a mild effect on cholinergic neurons, probably due to the fact that the BDNF decrease is downstream in the cascade altered by NGF deprivation and thus is not able to completely counteract the actions of anti NGF antibodies on basal forebrain cholinergic neurons, despite the fact that BDNF has been shown to induce their differentiation [66, 67] and protect these neurons from degeneration following axotomy [68]. BDNF genetic delivery has been shown to reduce the deficit in synaptophysin expression found in AD FAD models and also the rescue of synaptophysin expression in aged AD FAD mice obtained by neuronal stem cell transplant has been linked to increased BDNF brain content [16, 17]. Synaptic state would have been better assessed through the evaluation of multiple synaptic markers and different methodologies (confocal microscopy for synapse density and western blot for protein expression). It is a limit of our paper that drebrin was assessed only with western blot and synaptophysin only with immunohistochemistry and optical microscopy. However, our data show a decrease in drebrin and synaptophysin in AD11 mice which is not rescued by intranasal BDNF. This might result from the decrease in BDNF expression in AD11 mice being downstream to NGF deprivation [26] and, therefore, BDNF administration is not necessarily expected to rescue all effects of NGF deprivation.

In this study, as a first step to explore the possible involvement of brain inflammation in BDNF induced rescue of cognitive deficits in AD11 mice, we have assessed the presence of microgliosis in the hippocampus. We have observed a dramatic decrease of CD11b immunoreactive brain microglia. CD11b belongs to the family of integrins and is a subunit of the complement receptor 3. CD11b is known to play an important role in the adherence of neutrophils and monocytes to stimulated endothelium and in phagocytosis of complement coated particles [69]. In the brain, CD11b is expressed on microglia cells, its expression is up-regulated by reactive oxygen species [70] and it has been correlated, at least in vitro, with neuronal death during development [71] and in Parkinson’s disease models [72].

In the hippocampus, CD11b microglia has been found to be the major source of interleukin-1β [73], which, if produced in excess, contributes to neurodegeneration and to memory impairment [24, 74]. The ageing brain seems to be particularly sensitive to inflammation: indeed, a severe bacterial infection, which causes an increase in hippocampal IL-1β, compromises the cognitive status of aged rats more than in younger adult rats [24]. The authors concluded that the neuroinflammatory response is amplified in the aged brain, possibly due to sensitized microglia in ageing.

Previous indications already suggested that BDNF might reduce neuroinflammation. Physical exercise, which increases BDNF levels in the hippocampus and neocortex, reduced neuroinflammation and cognitive deficits in a model of stroke [75]. More importantly, Barrientos et al. [76] examined the effect of voluntary exercise in very old (24 months old) rats, subjected to bacterial infection-induced IL-1β increase in the hippocampus. The results show that exercise was sufficient to completely reverse infection-induced impairments in hippocampus-dependent long-term memory compared to sedentary animals. Interestingly, exercise prevented also the amplified neuroinflammatory response and the reduction in BDNF mRNA observed in the hippocampus of sedentary rats and strongly reduced age-associated microglial sensitization.

Microglia express both the phosphorylated and truncated forms of TrkB and can, therefore, respond to BDNF [77, 78]. BDNF has been reported to modulate local inflammation in ischaemic insults by modulating the activation of microglia in the brain after ischemic insults [60, 79]. The sole use of Cd11b does not allow to firmly establish that changes in activation only reflect activation of microglia cells, but only to suggest it, and to hypothesize that in AD11 mice BDNF administration may improve memory deficit by modulating microglia activity. Further studies are required to analyze the cytokine profile in BDNF –treated AD11 mice and provide insights into the BDNF-dependent signalling cascade that influences microglia activity and about the downstream neuroprotective and synaptoprotective consequences.

It has recently been shown that microglial and astroglial cells play a key role in the development and maintenance of brain inflammatory response in ageing and neurodegenerative diseases, showing enhanced proliferation and activation [25]. In the AD11 model, a dramatic neuroinflammatory response is among the earliest events triggered by anti NGF antibodies in the onset of the neurodegeneration progression [25]. Gomez Nicola et al. [80] studied the time course and regulation of microglial proliferation, using a mouse model of prion disease. They find that the proliferation of resident microglial cells accounts for the expansion of the population during the development of the disease and identify a pathway regulated by the activation of CSF1R as the molecular regulator of the proliferative response. In addition, they show that targeting the activity of CSF1R inhibits microglial proliferation and slows neuronal damage and disease progression, suggesting that microglial proliferation is a major component in the evolution of chronic neurodegeneration.

Recently, a reduced neurogenesis in AD11 mice has been observed, affecting both the dentate gyrus in the hippocampal formation [81] and the subventricular zone along the lateral walls of the lateral ventricles [82]. It is worth noticing that BDNF is a key molecule in mediating neurogenesis enhancement following Environmental Enrichment stimulation [83] and interestingly, BDNF overexpression and elevated neurogenesis have been proved to mimic the beneficial effects of physical exercise on cognition in 5xFAD mice [84]. In light of these observations, it is likely that the improvements in cognitive functions, that we observed in BDNF-treated mice may involve the enhancement of adult hippocampal neurogenesis. We did not perform a behavioural test specifically aimed at studying the effects of changes in hippocampal neurogenesis, (pattern separation tests). However, the results of the Object in Contest Test do suggest that BDNF intranasal infusion improved hippocampal functionality.

Our results provide evidence that non-invasive BDNF administration is effective in rescuing cognitive deficits in animal models of progressive neurodegeneration with Alzheimer-like characteristics and suggest that such cognitive improvement may be mediated by an improvement in hippocampal functionality, possibly related to an increase in hippocampal neurogenesis and a decrease in activated microglia. This reinforces the potential therapeutic uses of BDNF in neurological disorders [38], the potentiality of the non-invasive intranasal route as brain delivery strategy for BDNF or other neurotrophic factors and prospects microglia as a target cell population for BDNF neuro- and synapto-protective therapeutic actions.
